# Dietary enrichment of apolipoprotein E-deficient mice with extra virgin olive oil in combination with seal oil inhibits atherogenesis

**DOI:** 10.1186/1476-511X-10-41

**Published:** 2011-03-03

**Authors:** Karl-Erik Eilertsen, Hanne K Mæhre, Katrien Cludts, Jan O Olsen, Marc F Hoylaerts

**Affiliations:** 1 Norwegian College of Fishery Science, University of Tromsø, Tromsø, Norway; 2 MabCent-SFI, University of Tromsø, Tromsø, Norway; 3 Center for Molecular and Vascular Biology, Katholieke Universiteit Leuven, Leuven, Belgium; 4 HERG, Department of Medical Biology, Faculty of Health Sciences, Tromsø, Norway

## Abstract

**Background:**

In this study we investigated the antiatherogenic effect of dietary enrichment of a combination of extra virgin olive oil (EVO) and seal oil on apolipoprotein E-deficient (apoE^-/-^).

**Methods:**

Six-week-old female and male apoE^-/- ^mice were for 12 weeks fed a lipid rich diet containing 19.5% fat and 1.25% cholesterol without any supplement, with 1% (wt/wt) mixture of extra virgin olive and seal oil (EVO/n-3), or 1% corn oil, respectively.

**Results:**

Supplementation with the combination of EVO/n-3 significantly reduced atherosclerotic lesion formation in the aortic arch, thoracoabdominal, and total aorta of female apoE^-/-^mice. The effect was less pronounced in male mice and significant reduction was only observed in the thoracoabdominal region of the aorta. There were no differences or changes in dietary intake or body weight gain. However, compared to the other groups, plasma levels of triglycerides were reduced in both female and male mice fed the EVO/n-3 mixture. Male mice on both treatments showed reduced plasma cholesterol compared to the control mice after 12 weeks on diet.

**Conclusion:**

Dietary supplementation of a marine/olive oil combination inhibits atherosclerotic lesion formation in the female apoE^-/- ^mice by antithrombotic, antihypertriglyceridemic, and antioxidant effects.

## Introduction

Atherosclerosis is a disease with a multi-faceted aetiology, and diet is one of the most important environmental factors influencing the development of cardiovascular disease (CVD). The Mediterranean diet rich in extra virgin olive oil (EVO) has been associated with reduced rates of cardiovascular disease (CVD) [1-4]. EVO has a healthy fatty acid content with a high content of monounsaturated fatty acids (MUFA), mainly oleic acid (C-18:1n-9). Most other oils with a similar fatty acid composition are exposed to a refining process before consumption, in which presumably healthy components are removed or reduced. Unlike these, first-press EVO retains the important minor mainly phenolic compounds having properties considered to be anti-oxidant and anti-atherosclerotic [5]. Human consumption of EVO reduces major risk factors of atherosclerosis by improving the lipoprotein profile, blood pressure, glucose metabolism, and oxidative stress [6]. Similarly, considerable attention has been paid to the potential health benefits of the Greenland Inuit diet rich in fish, meat and blubber from marine mammals (seal and whale) resulting in a high dietary intake of long chain n-3 polyunsaturated fatty acids (PUFA), as this dietary regime also has been associated with a very low incidence of CVD [7]. PUFA has been considered as the major cardioprotective component in fish.

Research in our laboratories has previously demonstrated that co-supplementation of extra virgin olive oil and marine n-3 PUFA exerts anti-inflammatory effects by inhibiting lipopolysaccharide-induced monocyte activation *in vitro *[8,9], hence we hypothesized that a combination of EVO and seal oil should bring about a synergistic beneficial effect and reduce the development of CVD through prevention of oxidation of PUFAs and vascular inflammation. Intake of both marine PUFA and EVO has been associated with protection against coronary heart disease and sudden death. However, direct effects on atherosclerosis remain controversial. To that end, Apolipoprotein (apo) E-deficient mice (6 weeks) were fed an atherogenic Western type diet (high fat, high cholesterol) enriched with *i*) 1% of a combination of EVO and seal oil (EVO/n-3), *ii*) 1% corn oil (CORN) or *iii*) no supplement (WD) for 12 weeks to study the impact on plasma lipids and the development of atherosclerosis. ApoE-deficient mice spontaneously develop atherosclerosis with features similar to those observed in humans and is widely used to study the effect of diets on lipid metabolism and atherosclerosis [10,11].

## Methods

### Animals and diets

In total, 62 (30 females and 32 males, weighting 16.9 ± 1.2 g and 20.2 ± 1.2 g, respectively) ApoE^-/- ^mice (B6.129P2-Apoe^tm1UncN11^) 5 weeks of age obtained from Taconic (Taconic M&B, Ry, Denmark) were ear-marked and randomly allotted to three experimental groups, with equal numbers of cages in same-sex groups. This study was approved by the Institutional Animal Research Committee, and all experiments were performed following FELASA recommendation and according to the Belgian legislation on care and use of experimental animals. All animals were maintained in the same room at a room temperature of 21 ± 1°C and 50 ± 10% relative humidity, on a 12 h day/12 h night cycle (light on at 06:00 h) in a conventional laboratory animal unit. All animals were kept in type III Makrolon cages (Scanbur, Køge, Denmark) (37×21×15 cm). After one week of acclimation, the mice were fed a powdered atherogenic diet (1.25% cholesterol, 0% cholic acid, and 19.5% crude fat; ssniff Spezialdiäten GmbH, Soest, Germany) ad libitum for 12 weeks. The mice were divided into 3 groups of each sex and given the standard diet without any modification, or supplemented with 1% (w/w) mixture of EVO and seal oil (EVO/n-3; Olivita™, Olivita AS, Tromsø, Norway) or 1% (w/w) corn oil (CORN), respectively. The experimental diets were stored at 4°C, changed twice each week, and food consumption was recorded for every cage.

### Analysis of Atherosclerosis - Whole mount en face evaluation

After 12 weeks on the high-fat diet, mice were fasted for 4 h and anesthetized by intra-peritoneal pentobarbital (60 mg/kg pentobarbital; Nembutal, Abbott Laboratories), blood was drawn by retroorbital puncture, and the whole animal was perfused through the left ventricle with sterile saline (0.9%) for 5 min, followed by 1% paraformaldehyde (PFA) in PBS, pH 7.4 for 5 min. The entire aorta from the proximal ascending aorta to the bifurcation of the iliac arteries was dissected and cleaned *in situ *from the periadventitial tissue and opened longitudinally. After fixation in 1% PFA overnight, aortas were washed in PBS, soaked briefly in 78% methanol, stained with oil red O (ORO) for 30 min while shaking, and destained in 78% methanol for 2×5 min and mounted *en face *on slides under coverslips within Kaiser's glycerol gelatin (Merck, Darmstadt, Germany). ORO was prepared as a 0.2% stock in methanol and 35 ml stock was mixed with 10 ml of 1 M NaOH and filtered just before staining. Slides were allowed to dry for 2 days and the luminal side of the vessels were photographed using a Zeiss AxioPlan 2 imaging microscope equipped with a Zeiss AxioCam HrC camera. Photographs were evaluated for lesion area by morphometry and the extent of lesion development was reported as percentage of the total area of a given artery that was occupied by atherosclerotic lesions.

### Plasma Cholesterol and Triacylglycerol Levels

Mice were fasted for 4 hours, anesthetized with intraperitoneal pentobarbital (60 mg/kg Nembutal) before collection of venous blood from the retro-orbital sinus into a heparinised capillary tube. Fasting plasma samples were obtained for total cholesterol and triacylglycerol determinations at baseline, after 6 weeks on the experimental diet, and at the end of the study (12 weeks). Plasma was isolated by centrifugation at 3000 *g *for 10 minutes at 4°C and stored at -20°C. Enzymatic measurements of total cholesterol and triacylglycerol levels were performed using standard methods at the clinical chemistry department, UZ Leuven, Belgium.

#### Fatty acid composition of the diets

Dietary fatty acids (FA) was extracted and analyzed as described previously [12].

### Oxidation stability of the oils tested

An oxygen consumption method (Oxidograph, Mikrolab Aarhus, Brabrand, Denmark) was used to measure the in vitro oxidation stability of commercial a n-3 concentrate, seal oil, EVO, and a combination of seal oil and EVO (test conditions: temperature 70°C, sample 5 g of oil, rate 1 s-1) [13]. The induction period (hours) until the PUFA started to oxidize was measured to compare the oxidation stability of the tested oils. The Oxidograph measurements were duplicated and mean values with standard deviations were reported for each case.

### Statistical Analyses

All of the analyses were performed using SPSS for Windows (release 15.0.1; SPSS, Inc., Chicago, IL, USA). Unless otherwise stated, results are presented as mean ± standard deviation (SD). The Kolmogorov-Smirnov and Shapiro-Wilk tests were used to determine the distribution of the variables, and non-normally distributed variables were log transformed before statistical analysis. The significance of any differences between the groups at baseline or the impact of treatment on the changes in each group was determined using one-way analysis of variance (ANOVA) with post hoc comparisons of the variables using the Tukey's test. Within-group comparisons during the time-course of the experiment were performed using paired-group student's t-test or Mann-Whitney U-test where appropriate. Differences at the level of *P *< 0.05 were considered statistically significant.

## Results

### The antioxidant effect of EVO on oxidation of seal oil at 70°C

The oxidation stability of the PUFAs was determined using the Oxidograph apparatus. A longer induction period is an index for the resistance to oxidation. When the seal oil alone was subjected to oxidation at 70°C, the induction period was 2.1 hrs compared to 38.3 hrs for the combination of seal oil and EVO used in this study (values are average of 2 measurements). In comparison oxygen consumption started at 0.2 hrs for the commercial omega-3 concentrate.

#### Fatty acid composition of the diets

As shown in table 1, the PUFA enriched diets contained less saturated fatty acids (corn: 55.0 ± 0.2 g/100 g lipids, EVO/n-3: 55.0 ± 0.1 g/100 g lipids) compared to the control diet (56.5 ± 0.4 g/100 g lipids). The corn oil-enriched diet contained more C18:2n-6 linoleic acid (9.5 ± 0.1 g/100 g lipids) compared to the other experimental diets (control: 7.0 ± 0.1 g/100 g lipids, EVO/n-3: 6.9 ± 0.0 g/100 g lipids), whereas the content of linolenic acid (C18:3n-3) was identical between all diets (0.6 ± 0.0 g/100 g lipids). Compared to the other two diets the EVO/n-3 diet contained more MUFA, especially C18:1n-9 oleic acid (31.3 ± 0.0 g/100 g lipids vs control: 29.9 ± 0.2 g/100 g lipids, corn: 30.1 ± 0.1 g/100 g lipids), and finally the EVO/n-3 diet was the only diet containing detectable amounts of the marine n-3 fatty acids eicosapentaenoic acid; (EPA; C20:5n-3), docosapentaenoic acid (DPA; C22:5n-5), and docosahexaenoic acid (DHA; C22:6n-3).

**Table 1:** Fatty acid composition of the experimental diets (g/100g lipids; n = 3).

Fatty acids	Control	Corn oil	EVO^1^/n-3
C14:0	0.25 ± 0.01	0.28 ± 0.02	0.38 ± 0.02
C16:0	23.61 ± 0.20	23.32 ± 0.22	23.16 ± 0.03
C16:1n-7	0.23 ± 0.00	0.23 ± 0.00	0.73 ± 0.01
C18:0	31.45 ± 0.19	30.25 ± 0.09	30.26 ± 0.08
C18:1n-9	29.90 ± 0.24	30.12 ± 0.07	31.30 ± 0.03
C18:1n-7	0.54 ± 0.01	0.55 ± 0.01	0.66 ± 0.01
C18:2n-6	7.03 ± 0.08	9.49 ± 0.05	6.94 ± 0.01
C18:3n-3	0.63 ± 0.00	0.64 ± 0.00	0.64 ± 0.00
C20:0	1.00 ± 0.01	0.98 ± 0.01	0.97 ± 0.01
C20:1n-9	0.12 ± 0.00	0.10 ± 0.00	0.37 ± 0.03
C22:0	0.21 ± 0.00	0.20 ± 0.00	0.20 ± 0.00
C20:5n-3	nd	nd	0.18 ± 0.00
C22:5n-3	nd	nd	0.11 ± 0.00
C22:6n-3	nd	nd	0.23 ± 0.01

Σ saturated FA^2^	56.52 ± 0.36	55.03 ± 0.20	54.98 ± 0.09
Σ monounsaturated FA	30.70 ± 0.29	30.93 ± 0.08	33.06 ± 0.04
Σ polyunsaturated FA	7.66 ± 0.08	10.13 ± 0.05	8.10 ± 0.02
n-3 polyunsaturated FA	0.63 ± 0.00	0.64 ± 0.00	1.16 ± 0.02

^1^EVO, extra virgin olive oil. FA, fatty acids

### Mice growth and food intake

**Figure 1:**
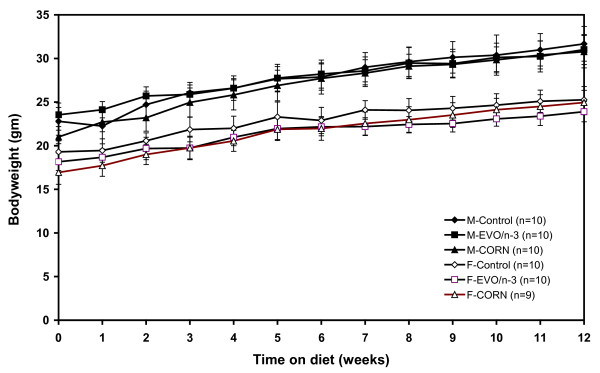
**Body weight curves for female and male apoE-deficient mice fed the experimental diets for 12 weeks**. Body weight was monitored each week. There were no differences in growth within each gender (ANOVA).

All the mice thrived and gained weight throughout the study. There were no apparent differences in growth between the different groups, or compared to mice kept on standard chow. Figure 1 shows the changes in body weight of the groups from the beginning to the end of the study.

### Plasma total cholesterol and triacylglycerol levels

Initial plasma total cholesterol and triglyceride concentrations were between 2.38 and 7.55 mmol/l in all groups at the start of the study (Table 2). The female mice fed EVO/n-3 had higher plasma total cholesterol levels at the start of the study compared to the CORN group (5.79 ± 1.20 vs 3.97 ± 0.63, p < 0.01), and after 6 weeks on diet plasma TC was higher compared to the control group (19.4 ± 3.70 vs 14.1 ± 3.67, p < 0.01). Both cholesterol and triacylglycerol levels tended to decrease towards the end of the intervention where plasma cholesterol was similar among the females. For all the groups fed the western-type diets (control, corn oil and EVO/n-3) a two-to-five-fold increased plasma TC (P < 0.05) compared to baseline cholesterol was observed, whereas plasma triacylglycerol levels generally decreased during the study period. In male mice, plasma TC increased significantly for all groups between the start and week 6. After 12 weeks on diet, plasma cholesterol was significantly lower for males fed either oil treatment compared to the controls. At the end of the 12-wk experiment, plasma TC for the chow-fed male mice was 6.95 ± 0.90 mmol/l.

**Table 2:** Plasma lipids in the six different study groups

		Cholesterol (mmol/l)			Triglycerides (mmol/l)		
		
		Week 0	Week 6	Week 12*	Week 0	Week 6	Week 12
Females							
No supplement	(n = 11)	4.93 ± 1.28	14.1 ± 3.67*^,a^	12.4 ± 1.63*	0.36 ± 0.10	0.28 ± 0.09	0.41 ± 0.45^a^
EVO^1^/n-3	(n = 11)	5.79 ± 1.20^a^	19.4 ± 3.70*^,b^	13.9 ± 3.76*	0.49 ± 0.27	0.32 ± 0.10	0.22 ± 0.03*^,b^
Corn oil	(n = 08)	3.97 ± 0.63^b^	15.9 ± 1.33*	14.1 ± 1.80*	ND	ND	ND
Males							
No supplement	(n = 11)	3.99 ± 1.26	16.1 ± 5.90*	20.2 ± 5.11*^,a^	0.42 ± 0.20	0.32 ± 0.16	0.46 ± 0.24
EVO/n-3	(n = 11)	4.57 ± 1.18	13.5 ± 2.62*	12.7 ± 4.08*^,b^	0.42 ± 0.14	0.25 ± 0.05*	0.23 ± 0.02*^,b^
Corn oil	(n = 10)	3.91 ± 0.60	15.0 ± 3.53*	14.7 ± 3.75*^,b^	0.27 ± 0.13	0.34 ± 0.12	0.51 ± 0.31^a^
Chow	(n = 05)	5.21 ± 1.55	ND	6.95 ± 0.90	0.53 ± 0.40	ND	0.38 ± 0.16

Cholesterol and triglyceride levels at the start of the study (week 0), after 6 weeks and at the end of the study (week 12). Values are mean ± S.D. Means within the same column bearing different superscripts are significantly different (p < 0.05 for individual comparison using the Tukeys' post hoc test after ANOVA). * Value significantly different from start concentration (week 0, P < 0.05) ^1^EVO, extra virgin olive oil. Significant differences between groups at certain timepoints are indicated with letters a and b.

### Atherosclerotic Lesions

**Figure 2:**
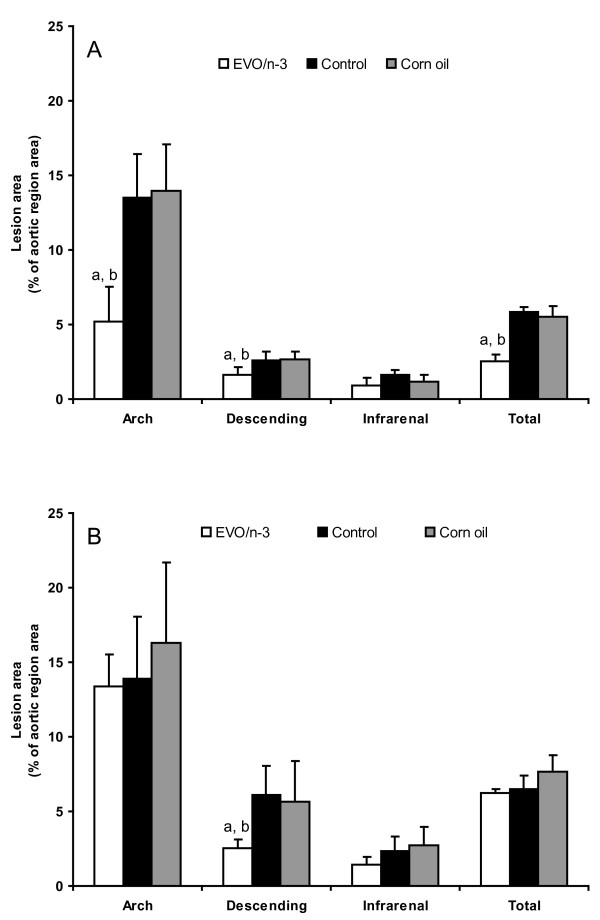
**Atherosclerotic plaque burden in the different aortic areas in female (A) and male (B) apoE-deficient mice**. Atherosclerosis is expressed as the % area covered by lipid Oil Red O staining. P-values are the result of post hoc Tukeys' test when the ANOVA was significant. ^a^atherosclerotic lesion is significantly different than control group (P < 0.01), and ^b^lesion is significantly different from corn oil group (P < 0.01). EVO, extra virgin olive oil.

The influence of the combination of EVO and seal oil on the development of atherosclerosis was analyzed in both female and male apoE^-/- ^mice. *En face *atherosclerotic lesions in the opened aortas were evaluated as percent area positive to lipid ORO-staining both in the whole aorta and regionally, calculating the percent area staining red in the aortic arch, in the thoracoabdominal part of the descending aorta and in the infrarenal part down to the iliac bifurcation. The lesions were mainly distributed in the aortic arch and the areas surrounding the branching points of the arteries. A significant plaque reduction was observed in the aortas from the EVO/n-3-fed mice compared to both the other dietary groups. This effect was particularly pronounced for the female mice (Figure 2), where the plaque burden in the aortic arch of the EVO/n-3-group (5.2 ± 2.3%) were reduced by 61% (P < 0.001) and 63% (P < 0.001) compared to the control group (13.5 ± 2.9%) and the CORN-enriched group (13.9 ± 3.1%), whereas there were no significant differences between the control group and the CORN-enriched group. Also in the thoracoabdominal region of the aorta, (EVO/n-3 1.60 ± 0.53%, control group 2.62 ± 0.57%; P < 0.01, and corn oil 2.67 ± 0.52%; P < 0.01), and in the entire aorta subjected to analysis, (EVO/n-3 2.54 ± 0.45%, control group 5.85 ± 0.35%; P < 0.01, and CORN 5.55 ± 0.67%; P < 0.01), enrichment with the EVO/n-3 combination significantly reduced atherogenesis. In the infrarenal region (EVO/n-3 0.88 ± 0.58%, CORN 1.17 ± 0.48%, and control group 1.62 ± 0.33%), the reduction was not significant.

In the male mice, differences were only weak, and the only significant reduction were observed in the thoracoabdominal region of the aorta, were the plaque burden were significantly less in the male apoE-deficient mice fed diets enriched with the combination of EVO/n-3, compared to the control mice. There were no visible atherosclerotic lesions in any area of the aortas from the chow (standard low-fat maintenance mouse feed) fed apoE-deficient male mice (data not shown).

## Discussion

The purpose of this study was to investigate the effect of dietary supplementation of apoE-deficient mice with EVO in combination with seal oil on atherosclerotic lesion formation. The rationale for combining the refined marine oil with EVO high in antioxidative phenolic compounds was to combine the beneficial effects of both these dietary oils, and furthermore to prevent the potential harmful effects related to oxidation of the highly unsaturated n-3 fatty acids which is likely to occur in milieus having low levels of antioxidants. In addition, it has previously been observed that some beneficial effects from crude marine oils are lost after refinement [14]. Improved anti-inflammatory and anti-platelet effects of dietary supplementation with a cod liver (CLO)/olive oil blend compared to separate supplementation with these oils have previously been reported [8].

In support of our hypothesis, female mice fed diets containing EVO/n-3 had reduced atherosclerotic lesion formation throughout the entire aorta, whereas male mice on the same diet only had a significantly reduced lesion formation only in the abdominal region of the aorta. The weaker response in male mice may be explained by the atheroprotective effect of estrogen [15], which reduce the atherosclerotic pressure and may cause a situation where the effect of the supplementation in combination with a potential gender associated estrogen effect results in reduced atherogenesis. Evidence for an estrogen-dependent mechanism in the regulation of atherogenesis was recently reported in apoE^-/-^-mice mediated via the heat shock protein 27 [16]. Thus, modulation of the release of this anti-atherogenic protein may be one possibility for the effect of the test oil in female mice. Other gender differences have been associated with lesion formation in apoE^-/-^-mice [17] and in a recent study; gender-specific n-3 PUFA-related antiplatelet effects were observed when healthy subjects were given EPA or DHA [18]. However, even when results from both genders were combined, dietary supplementation with EVO/n-3 significantly reduced aortic lesion formation.

Lipid peroxidation plays an important role in atherogenesis and the anti-atherogenic effect observed in the EVO/n-3 group may at least partly be attributed to the antioxidant compounds inherent in the cold pressed olive oil [5,19,20]. EVO clearly protects the n-3 PUFA against oxidation as the oxidograph experiments demonstrate that the blend of EVO/n-3 is oxidized at 70°C after 38 hrs compared to 0.2 hrs for the n-3 PUFA concentrate and 2.1 hrs for seal oil alone.

Thus, the EVO part of the EVO/n-3 combination comprises potent antioxidants, particularly polyphenols that may prevent the oxidation of the PUFAs and thereby abolish modification of LDL-cholesterol in the intima and subsequent oxidative stress [20]. Indeed, the amount of polyphenols in olive oil has been demonstrated to be correlated to both increases in HDL-cholesterol and decreases in oxidative stress markers in healthy humans [21]. The EVO antioxidants, which probably play a central role in the investigated oil combination, have recently been documented to contribute to the health benefits derived from the Mediterranean diet [5]. In addition, EVO and polyphenol-enriched EVO have been demonstrated to moderately reduce atherosclerosis in ApoE-deficient mice [22]. In combination with seal oil we suggest that these beneficial effects may be accelerated. Interestingly, the combined oil product of EVO/n-3 induces a strong stinging sensation in the throat, associated with the presence of the anti-inflammatory compound oleocanthal from the EVO [23]. The present study indicated that marine oils might contain other beneficial components of relevance for CVD in addition to n-3 PUFA. Similarly, the combination of seal oil and cold pressed olive oil as used in this study, was found to have superior beneficial effects (anti-inflammatory and rise in HDL-cholesterol) compared to CLO that contained much higher levels of n-3 PUFA in a clinical study of healthy individuals [9]. Our studies suggest that by recombining refined seal oil, void of antioxidants (removed through refinement) and other contaminants, with extra virgin olive oil, a synergistic effect is obtained between the marine PUFAs, particularly EPA and DHA, and the powerful minor antioxidant compounds in the olive oil, in addition to the high concentration of the monounsaturated oleic acid (n-9) inherent in the EVO.

As generally recognized, dietary supplementation with n-3 PUFA, here in combination with extra virgin olive oil, reduced plasma triacylglycerol levels. In addition, EVO/n-3 supplementation seemed to reduce plasma total cholesterol. However, this effect was more pronounced in the male mice, therefore the prevention of atherogenesis observed in the EVO/n-3-enriched group probably reflects the beneficial anti-inflammatory effect previously observed in healthy humans where intake of 15 ml/day in 10 weeks of EVO/seal oil caused a mean reduction of 14.3% in monocyte chemotactic protein-1 (MCP-1) and 24.0% hypersensitive CRP (hsCRP) compared to respectively 5.2% and an increase of 12.5% for intake of 15 ml fish oil (Østerud, personal communication). The corn oil diet contains significantly more 18:2n-6, compared to the other two diets, and improved n-3/n-6 ratio caused by intake of n-3 PUFA from the seal oil and less n-6 PUFA, are also considered beneficial [12].

Direct anti-atherosclerotic activities of n-3 PUFA remains to be demonstrated, however, several lines of evidence suggest an beneficial effect of increased intake of n-3 PUFA. Dietary n-3 PUFA (EPA, DPA, and DHA) are incorporated into cell membranes and an important effect of increased intake of n-3 PUFA is reduced release of arachidonic acid and subsequent production of its proinflammatory metabolites prostaglandins, leukotrienes and thromboxanes [24]. Recently, administration of DHA was demonstrated to inhibit the activity of secretory phospholipase A_2 _as well as to reduce the production of superoxide via inhibition of NADPH oxidase in endothelial cell cultures [25]. In addition, n-3 PUFA compete with n-6 PUFA for access to receptors and enzymes such as lipoxygenases and cyclooxygenases, further reducing the potential in the circulation for activation of circulating platelets and other immunomodulating cells. Both TxA_2 _and the 5-lipoxygenase pathway/LTB_4 _have been indicated to be significant contributors to atherogenesis in apoE-deficient mice [26-29]. In this regard, the relatively high concentration of DPA in seal oil compared to fish oil may at least partially account for the atheroprotective effect of EVO/seal oil as it has been shown that DPA is more potent than EPA and DHA in inhibiting TxA_2 _formation. Furthermore, DPA has been shown to reduce platelet activation in response to collagen or arachidonic acid via enhanced formation of 12-HETE [30]. It has previously been demonstrated that the biological effects of marine oils do not directly correlate to the total content of n-3 PUFA. Accordingly, intake of cold pressed whale oil for 10 weeks in human healthy volunteers resulted in beneficial effects such as rise in HDL-cholesterol, reduced generation of pro-inflammatory products in LPS-stimulated blood that was superior to regular CLO despite the 50% lower content of n-3 PUFA, but higher DPA content, in the whale oil as compared to CLO [14]. Finally, it is evident that dietary EPA is incorporated into advanced atherosclerotic lesions resulting in decreased intravascular inflammation and increased plaque stability [31,32].

## Conclusions

This study demonstrates that EVO/n-3 supplementation reduces atherosclerotic lesion formation, particularly in female apoE-deficient mice. The antiatherogenic effect of the EVO/n-3 was to some extent accounted for by lowering plasma total cholesterol and triacylglycerol levels. The EVO rich in oleic acid and phenolic antioxidants appears to work well in combination with a marine n-3 PUFA source providing atheroprotective properties. This study needs to be followed up by experiments exploring the biochemical and cellular mechanisms responsible for the observed antiatherogenic effect.

## Authors' contributions

All authors contributed to the intellectual development of this study, and approved the final manuscript. KEE conceived the study, participated in experimental design, prepared the diets, carried out the animal experiments, did the sampling and prepared the samples, performed the lesion analyses and statistical analysis, and drafted the manuscript. HKM carried out the fatty acid analyses and provided critical corrections to the manuscript. KC handled the animals, prepared and handled the diets and prepared samples, MFH conceived the study, participated in experimental design, contributed with experimental expertise and provided critical corrections to the manuscript. JOO carried out lesion analyses.

## Declaration of competing interests

The authors declare that they have no competing interests.
